# Change of luminal diameter of skeletonized and non-skeletonized radial artery graft at early and late postoperative period

**DOI:** 10.1007/s00380-015-0639-3

**Published:** 2015-02-06

**Authors:** Takuto Maruyama, Hiroki Kohno, Keiichi Ishida, Toru Ishizaka, Nobusada Funabashi, Yoshio Kobayashi, Goro Matsumiya

**Affiliations:** 1 Department of Cardiovascular Surgery, Chiba University Graduate School of Medicine, 1-8-1 Inohana, Chuo-ku, Chiba, Chiba 260-8670 Japan; 2 Department of Cardiovascular Science and Medicine, Chiba University Graduate School of Medicine, Chiba, Japan

**Keywords:** CABG, Radial artery, Luminal diameter, Skeletonization, Ultrasonic scalpel

## Abstract

The radial artery is increasingly used as a second arterial conduit for myocardial revascularization. However, the radial artery is susceptible to vasospasm, which is thought to be the principal cause of graft failure. The radial artery is harvested as a skeletonized or a non-skeletonized graft, but the effect of different harvesting technique remains unknown. In this study, we compared the early- and mid-term angiographic findings to elucidate its influence on the graft luminal diameter. We harvested 39 radial arteries either as a skeletonized (*n* = 18) or a non-skeletonized graft (*n* = 21) using an ultrasonic scalpel. We constructed a composite straight graft by combining a right internal thoracic artery and a radial artery. All the radial artery grafts were sequentially anastomosed to coronary arteries. We measured the diameters of the radial arteries before the operation, within 1 month and 1 year after the operation. At early postoperative period, graft diameter was significantly larger in skeletonized grafts. Graft diameter at the point before the first and the second anastomosis was similar in skeletonized grafts, although that was significantly smaller before the second anastomosis in non-skeletonized grafts. However, 1 year after the operation, the graft diameter was comparable and equally reduced after the first anastomosis in both groups. Skeletonization with an ultrasonic scalpel increases the luminal diameter of the radial artery graft at early postoperative period, which, however, reduces possibly as adaptation to graft flow 1 year after the operation.

## Introduction

Arterial grafts in coronary artery bypass grafting (CABG) are increasingly utilized since the superior long-term patency of internal thoracic artery (ITA) grafts was reported [1]. Currently, the radial artery (RA) has become the second choice of arterial conduits following ITA. Use of RA together with bilateral ITAs enables total arterial revascularization and possible avoidance of poor long-term patency of saphenous vein graft (SVG) [2, 3].

Although recent reports have shown better outcomes of RA grafts in comparison to SVG, most of those demonstrated its inferiority to ITA [4]. Prolonged ITA graft patency is related to its unique pathophysiological features including less muscular and more elastic components of media. On the contrary, RA is a muscular artery and susceptible to vasospasm, which is thought to be the principal cause of graft failure [5]. Therefore, it is of critical importance to prevent spasm of RA grafts. Besides pharmacological prophylaxis using systemic vasodilators or topical agents during graft harvesting, a harvesting technique has been considered to have significant influence on spasm of RA grafts [6]. Harvesting as a traditional “non-skeletonized” graft including an accompanying vein and surrounding connective tissue minimizes surgical trauma, thus was considered to reduce tendency to spasm [7]. However, recent studies reported the other harvesting technique “skeletonization” reduced spasm and improved RA graft patency [8, 9, 10]. These conflicting reports prompted us to examine the effect of the different harvesting technique on luminal diameter of the RA graft.

The aims of this study are to compare the early- and mid-term angiographic findings of the RA graft harvested either as a skeletonized or a non-skeletonized graft and examine the influence of the harvesting technique on the luminal diameter of the RA graft.

## Materials and methods

### Patients

Between July 2008 and September 2009, 39 patients underwent CABG using a straight composite graft constructed with a right internal thoracic artery (RITA) and RA. They underwent postoperative angiography within 1 month after the operation. In 18 patients RA was harvested as a skeletonized graft (Group S) and in the other 21 patients RA was harvested as a non-skeletonized graft (Group N) depending on operators’ preference. All patients were given informed consent about the study. Data were retrospectively collected, from medical records and image servers. Therefore, approval from our hospitals’ institutional review boards was unnecessary.

### Radial artery harvesting

All patients had preoperative examination of the RA by a duplex scan and the preoperative diameter of the RA (preRAD) was determined at 10 mm distal from its proximal end. The RA was harvested from the arm of the non-dominant hand. Skin incision was made from the wrist to the mid-antecubital fossa. RAs were harvested either as a skeletonized or a non-skeletonized graft. In both methods, an ultrasonic scalpel (Harmonic Scalpel; Ethicon Endo-Surgery, Cincinnati, OH) was used for harvesting. The branches of the RA were cut with protein coagulation by ultrasonic scalpel, and surgical clips were applied only to major branches when necessary in both procedures. For skeletonization of the RA, we incised the fascia covering the RA entirely with scissors at first, and the space between the RA and satellite veins was dissected using an ultrasonic scalpel. A non-skeletonized RA was dissected together with a satellite vein and surrounding connective tissue and finally the back of covering fascia was incised longitudinally with scissors. In both methods, the RA was wrapped with gauze soaked with phosphodiesterase inhibitor solution (olprinone hydrochloride, 12.5 mg/dl) until it was removed. After harvesting, the RA graft was flushed and stored in the same olprinone hydrochloride solution.

### Operation and postoperative management

After harvesting the RA, CABG was performed with or without cardiopulmonary bypass. In all the patients, the left internal thoracic artery (LITA) was anastomosed to the left anterior descending artery. RA was anastomosed with a skeletonized RITA as a composite straight graft. We anastomosed both grafts with minimal cut-back at the end of RITA graft. We did not observe any stenosis or kinking at anastomosed site in all patients. RA was sequentially anastomosed to more than 1 native coronary artery segment (the diagonal artery, the circumflex artery, or the right coronary artery) with diamond fashion. Severity of angiographic stenosis of the first and the second target vessel was not significantly different between the groups. We started continuous infusion of Nicorandil during graft anastomosis and continued in ICU, which was switched to oral administration of Isosorbide Mononitrate, once oral intake became possible. Calcium antagonist was not routinely used.

### Follow-up and angiographic studies

Postoperative angiography was performed within 1 month after the operation. The postoperative diameter of the RA was measured at 10 mm distal from the anastomotic site with the RITA (postRAD), at 10 mm proximal to the first (RAD1) and the second (RAD2) anastomotic sites, respectively. The postoperative diameter of the RITA was measured at 10 mm proximal from the anastomotic site with the RA (RITAD). These angiographic vessel diameters were calculated using the size of a catheter in the same field as a reference. Flow competition was defined as a situation in which the target branch was slightly opacified or was not opacified from the ITA injection, and the bypass graft was opacified by retrograde flow from the native coronary injection.

Twenty-seven patients (15 patients in Group S and 12 patients in Group N) underwent follow-up 320-slice CT angiography (Aquilion One, Toshiba Medical) 1 year after the operation. Retrospective ECG-gated conventional enhanced 320-slice CT was performed with a slice thickness of 0.5 mm. Contrast material was administered into the right antecubital vein. All patients received 10 mg of propranolol prior to scanning, except for those who had severe heart failure, bronchial asthma, hypotension, or bradycardia due to conditions such as atrioventricular block or sick sinus syndrome. Patients were excluded from this procedure if they did not provide consent or had renal failure. Evaluation of the RA on CT angiogram was performed using a workstation (ZIO M900, Amin/ZIO) [11].

### Statistical analysis

Data are expressed as mean ± SD. Risk factors, patency rates, and other parametric data were examined with contingency tables, Fisher’s exact test, the Chi-squared test, or Student’s *t* test as appropriate. Differences were considered significant with *P* < 0.05. All statistical analyses were performed using the JMP, version 11.0.0 (SAS Institute Inc., Cary, NC).

## Results

There was no statistically significant difference in the two study groups with respect to variables that are known to influence operative outcomes (Tables 1, 2). There was no in-hospital mortality or perioperative myocardial infarction. There was no complication associated with RA harvesting in both groups.

**Table 1:** Preoperative characteristics of patients

	Group S (*n* = 18)	Group N (*n* = 21)	*P* value
Male/female	14/4	18/3	0.52
Mean age (years)	65.6	66.3	0.82
Low ejection fraction (LVEF <35 %)	1	2	0.64
Previous myocardial infarction	10	9	0.43
Diabetes	11	10	0.40
Hypertension	17	19	0.64
Hyperlipidemia	11	15	0.50
Smoking	11	17	0.17
Peripheral vascular disease	2	3	0.77
Chronic renal dysfunction (Cr >1.5 mg/dl)	0	3	0.23
IABP	2	0	0.21

*LVEF* left ventricular ejection fraction, *Cr* creatinine, *IABP* intra-aortic balloon pumping

**Table 2:** Operative details

	Group S (*n* = 18)	Group N (*n* = 21)	*P* value
Total no. of RA anastomoses	45	57	
RA anastomoses per patient	2.5 ± 0.62	2.7 ± 0.56	0.26
Target location			
Dx	9 (20.0 %)	11 (19.3 %)	0.93
OM	6 (13.3 %)	12 (21.1 %)	0.31
PL	15 (33.3 %)	11 (19.3 %)	0.11
PD	15 (33.3 %)	22 (38.6 %)	0.58
S1	88 ± 7.0 %	87 ± 8.5 %	0.60
S2	93 ± 8.0 %	87 ± 9.7 %	0.08
CPB	3	0	0.09

*RA* radial artery, *Dx* diagonal artery, *OM* obtuse marginal artery, *PD* posterior descending artery, *PL* posterolateral artery, *S1*, *S2* % Stenosis of the target coronary artery of the 1st and 2nd radial artery anastomosis in preoperative coronary angiogram, *CPB* cardiopulmonary bypass

### Early angiographic results

On angiography at early postoperative period, two grafts were occluded in Group S and all the grafts were patent in Group N. Two grafts showed string sign in Group N, but there was no string sign in Group S. Flow competition was seen less often in Group S although that did not reach statistical significance. Perfect patency rate (patency rate without stenosis >50 % or flow competition) was not significantly different between the groups (Table 3).

**Table 3:** Early coronary angiographic results

	Group S (18 patients, 45 anastomoses)	Group N (21 patients, 57 anastomoses)	*P* value
Graft occlusion	2 (4.4 %)	0 (0.0 %)	0.19
String	0 (0.0 %)	2 (3.5 %)	0.50
Flow competition	6 (13 %)	12 (21 %)	0.31
Perfect patency	37(82 %)	45(79 %)	0.68
preRAD (mm)	3.00 ± 0.53	3.25 ± 0.65	0.20
postRAD (mm)	2.66 ± 0.59	2.40 ± 0.45	0.12
postRAD/preRAD	0.90 ± 0.19	0.75 ± 0.16	***0.02***
RITAD (mm)	1.93 ± 0.74	1.91 ± 0.67	0.89
post RAD/RITAD	1.4 ± 0.58	1.26 ± 0.53	0.25
RAD1 (mm)	2.64 ± 0.54	2.42 ± 0.41	0.15
RAD2 (mm)	2.61 ± 0.53	2.00 ± 0.58	***0.0022***
RAD2/RAD1	0.99 ± 0.034	0.83 ± 0.18	***0.0002***

Bold and italic values indicate *P* < 0.05

*preRAD* diameter of the preoperative radial artery measured by a duplex scan, *postRAD* diameter of the postoperative radial artery graft, *RITAD* diameter of the postoperative right internal thoracic artery graft, *RAD1* diameter of the radial artery at 10 mm before the first anastomosis, *RAD2* diameter of the radial artery at 10 mm before the second anastomosis

**Fig. 1:**
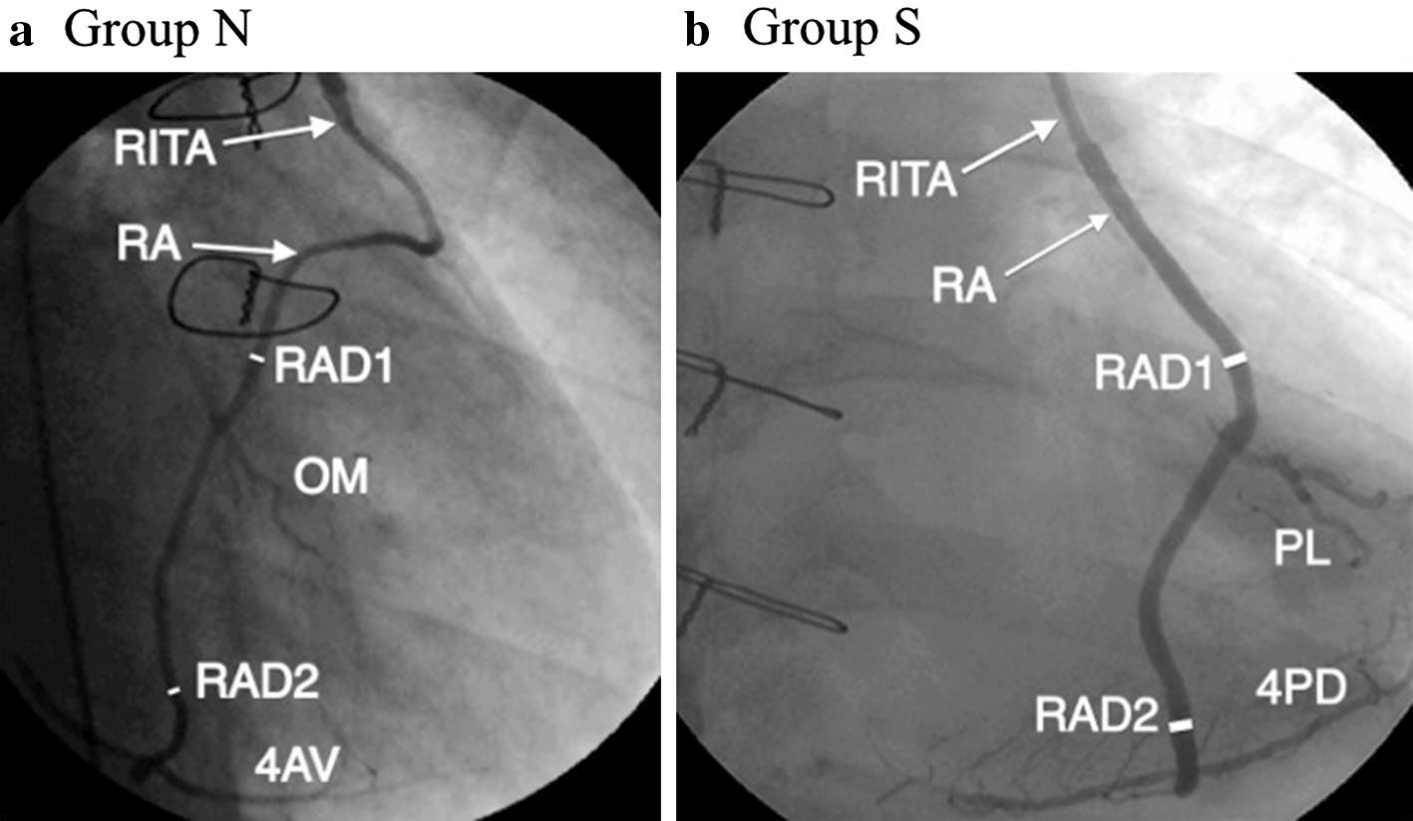
Postoperative coronary angiogram of composite straight grafts performed within 1 month after the operation. **a** Angiogram of a composite straight graft with a *right* internal thoracic artery and a non-skeletonized radial artery sequentially anastomosed to *OM* and *4AV*. The graft diameter before the second anastomosis (*RAD2*) was smaller than that before the first anastomosis (*RAD1*). **b** Angiogram of a composite straight graft with a *right* internal thoracic artery and a skeletonized radial artery sequentially anastomosed to *PL* and *4PD*. A skeletonized radial artery graft diameter was larger than a non-skeletonized radial artery graft. The radial artery did not change its diameter before the first (*RAD1*) and second anastomosis (*RAD2*). *4AV* atrioventricular node branch, *OM* obtuse marginal artery, *4PD* posterior descending artery, *PL* posterolateral artery, *RA* radial artery, *RAD1* diameter of the radial artery before the first anastomosis, *RAD2* diameter of the radial artery before the second anastomosis, *RITA* right internal thoracic artery

**Fig. 2:**
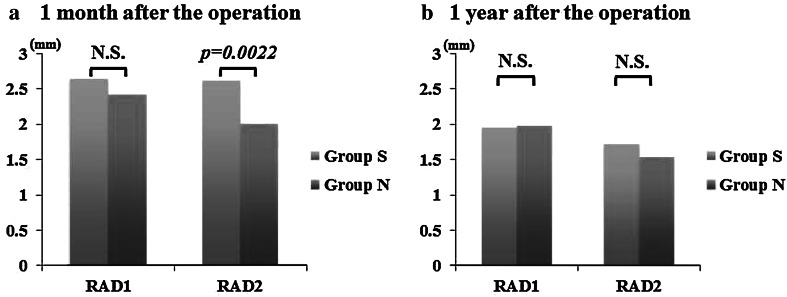
Periodical change of the diameter of radial artery graft before the first (RAD1) and the second anastomosis (RAD2). **a** The diameter of radial artery graft on postoperative coronary angiogram performed within 1 month after the operation. There was no significant difference in RAD1 between Group S and Group N. But RAD2 was significantly greater in Group S. **b** The diameter of radial artery graft on 320-slice CT angiography 1 year after the operation. There was no significant difference in RAD1 between Group S and Group N. And there was no significant difference in RAD2 between Group S and Group N. *RAD1* diameter of the radial artery graft at 10 mm before the first anastomosis, *RAD2* diameter of the radial artery graft at 10 mm before the second anastomosis

The mean diameter of the RA on angiogram at early stage after the operation (postRAD) was not significantly different between the two groups. However, the relative size to the preoperative diameter of the RA (postRAD/preRAD) was significantly larger in Group S (Table 3, Fig. 1). There was no significant difference in RAD1 between the two groups. But RAD2 was significantly greater in Group S. Therefore, the mean ratio of RAD1 to RAD2 on angiography (RAD2/RAD1) was significantly larger in Group S (Table 3, Figs. 1, 2).

### Results of 320-slice CT angiography 1 year after the operation

**Fig. 3:**
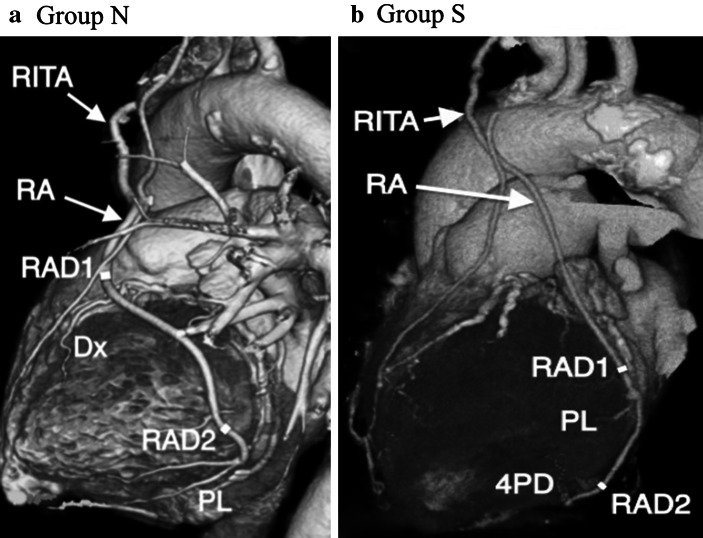
320-slice CT angiogram of composite straight grafts performed 1 year after the operation. **a** 320-slice CT angiogram of a composite straight graft with a *right* internal thoracic artery and a non-skeletonized radial artery sequentially anastomosed to *Dx* and *PL*. The graft diameter before the second anastomosis (*RAD2*) was smaller than that before the first anastomosis (*RAD1*). **b** 320-slice CT angiogram of a composite straight graft with a *right* internal thoracic artery and a skeletonized radial artery sequentially anastomosed to *PL* and *4PD*. The graft diameter before the second anastomosis (*RAD2*) got smaller than that before the first anastomosis same (*RAD1*). This finding is not consistent with that in coronary angiogram at early postoperative period. *Dx* diagonal artery, *PL* posterolateral artery, *4PD* posterior descending artery, *RA* radial artery, *RAD1* diameter of the radial artery before the first anastomosis, *RAD2* diameter of the radial artery before the second anastomosis, *RITA* right internal thoracic artery

On 320-slice CT angiography 1 year after the operation, frequency of graft occlusion or string sign was not significantly different between the two groups (Table 4). The significant difference on angiography at early stage after the operation in postRAD/preRAD, RAD2, and RAD2/RAD1 between the groups was disappeared on 320-slice CT angiography 1 year after the operation (Table 4, Figs. 2, 3). Skeletonized and non-skeletonized RA grafts had comparable diameter and similarly changed its diameter after the first anastomosis.

**Table 4:** Results of 320-slice CT angiogram 1 year after the operation

	Group S (15 patients, 39 anastomoses)	Group N (12 patients, 31 anastomoses)	*P* value
Graft occlusion	2 (5.1 %)	3 (9.7 %)	0.65
String	2 (5.1 %)	3 (9.7 %)	0.65
Perfect patency	35 (90 %)	25 (81 %)	0.28
preRAD (mm)	2.99 ± 0.56	3.43 ± 0.77	0.10
PostRAD (mm)	1.95 ± 0.46	1.98 ± 0.80	0.93
postRAD/preRAD	0.67 ± 0.15	0.58 ± 0.21	0.22
RITAD (mm)	1.78 ± 0.28	1.71 ± 0.53	0.78
post RAD/RITAD	1.10 ± 0.25	1.15 ± 0.23	0.73
RAD1 (mm)	1.95 ± 0.46	1.98 ± 0.80	0.91
RAD2 (mm)	1.72 ± 0.49	1.53 ± 0.93	0.49
RAD2/RAD1	0.88 ± 0.18	0.72 ± 0.39	0.16

*preRAD* diameter of the preoperative radial artery measured by a duplex scan, *postRAD* diameter of the postoperative radial artery graft, *RITAD* diameter of the postoperative right internal thoracic artery graft, *RAD1* diameter of the radial artery graft at 10 mm before the first anastomosis, *RAD2* diameter of the radial artery graft at 10 mm before the second anastomosis

## Discussion

The significant findings of this study are as follows. (1) In the early stage after the operation, skeletonized RA grafts maintained larger diameter in compared to non-skeletonized grafts. Unlike non-skeletonized grafts, skeletonized grafts did not show remodeling of the graft diameter. (2) In the mid-term follow-up CT angiogram, the skeletonized RA graft changed its diameter. There was no difference in this remodeling capacity between the skeletonized and the non-skeletonized graft.

Our first finding is consistent with several past reports. Yamaguchi et al. [12] reported that on angiography at an early postoperative stage, the mean diameter of the RA was wider and spasm or stenosis was seen less often in skeletonized grafts. Although the underlining mechanism for larger diameter and less vasoreactivity of skeletonized RA remains unknown, there are several possible explanations. First explanation relates to the use of an ultrasonic scalpel as a harvesting tool. Erkut et al. [13] and Ronan et al. [14] reported that ultrasonic dissection of RA has a positive effect on endothelial preservation and is associated with increased free blood flow through the graft. Maruo et al. [15] reported that sonication on canine ITA with an ultrasonic scalpel induces vasorelaxation almost completely by endothelial nitric oxide (NO) and prostacyclin release. In our study, we harvested the RA either as a skeletonized or a non-skeletonized graft, but used an ultrasonic scalpel in both groups. However, the sonication may have more directly and effectively stimulated endothelium in the skeletonized artery as it lacks surrounding tissue outside the adventitia. Second, RA is known to consist of relatively thick media packed with smooth muscle cells [5]. RA smooth muscle cells have higher receptor-mediated contractility in compared to ITA. RA is known to be an α-adrenergic receptor dominant artery with weak β-adrenergic receptor function [16]. RA exhibits greater contraction to several chemical mediators such as serotonin, thromboxane A2, and norepinephrine than ITA [16]. Skeletonization may influence the sensitivity of the receptor of the RA or release of mediator from the endothelium. Further physiological and pharmacological examination is required to elucidate the effect of skeletonization of RA graft.

It has been well known that arterial grafts change its diameter depending on flow requirement [17]. The present study demonstrated first time that this remodeling capacity differs depending on the harvesting technique. Skeletonization impaired the remodeling capacity in the early phase but that is regained in the later phase. The remodeling response largely depends on endothelial function that tries to maintain shear stress in the vessel constant. NO released from endothelium plays a crucial role in the remodeling of vascular diameter depending on shear stress [18]. Fukui and colleagues [19] reported that a non-skeletonized RA graft adjusts its caliber to suit the flow requirement, which eventually normalizes shear stress at the endothelial interface and allows the conduit to deliver an appropriate flow. Low shear stress induces the attachment of endothelial cells and leukocytes, resulting in thrombus, intimal hyperplasia, and subsequent graft failure [20]. If the postoperative diameter of the RA did not reduce to match the flow requirement, shear stress would be reduced as a result of the decreased flow velocity, which would eventually result in graft failure. In our study, the reduced vasoreactive capacity to the blood flow of the skeletonized radial artery graft seems to be recovered 1 year after the operation. The recovered vasoreactivity may contribute to avoid graft failure caused by the lack of the caliber change in response to the blood flow, although it is hard to justify the speculation from our study.

There are several points that could have influenced our results. First, RA straight composite graft with ITA can behave differently from that used as aortocoronary bypass. Composite grafting allows an aorta no touch technique when combined with off pump CABG, which has major advantage of decreasing perioperative stroke. On the contrary, decreased drive pressure in the composite graft compared to direct aorta anastomosis could compromise the flow through the graft [21, 22]. The opposite argument relates to the fact that ITA has increased capacity to release NO compared to other grafts [23], and thus may have rather beneficial impact on RA graft, which was anastomosed at its downstream. Currently, advantage of the straight composite graft is still unclear.

Second, multiple sequential grafting may also affect the fate of arterial grafts. There are conflicting reports on graft patency between sequential and single RA grafting. Nakajima et al. [24] reported that graft patency and competitive flow of sequential RA grafts is influenced by the severity of coronary artery stenosis in the most distal target artery. We preferred to use multiple sequential grafting to achieve complete arterial revascularization. This grafting strategy may have changed reaction of RA grafts.

Third, it has been well known that arterial grafts change its diameter depending on severity of target vessel stenosis [25]. In our study, severity of the target vessel stenosis was comparable between each group. However, visual assessment of the percentage of stenosis at angiography does not necessarily reflect accurate flow disturbance. Current studies demonstrated that fractional flow reserve (FFR) is more predictive of graft patency [26]. Our study does not include the assessment of FFR, thus flow requirement or flow competition of target vessel could have been different in each group.

This study has several limitations. First, the number of the patients in each cohort was small and selection of each harvesting methods was not randomized. Although the patient backgrounds were not significantly different between the groups, there was possible selection bias. Second, we could not assess several patients 1 year after the operation. Therefore, we emphasize the need for a large, randomized study.

In conclusion, at an early stage after the operation, the RA graft skeletonized with an ultrasonic scalpel maintains larger diameter irrespective of graft flow. It may contribute to avoid graft failure or flow competition in early postoperative period, when the grafts are especially susceptible to spastic stimuli. However, 1 year after the operation, luminal diameter of the skeletonized radial artery graft reduces possibly as physiological adaptation to graft flow. Further studies will be needed to confirm whether those characteristics of skeletonized radial artery graft result in any beneficial short- and long-term graft function.

## Abbreviations

CABG: Coronary artery bypass grafting
CT: Computed tomography
ITA: Internal thoracic artery
LITA: Left internal thoracic artery
NO: Nitric oxide
postRAD: Diameter of the postoperative radial artery
preRAD: Diameter of the preoperative radial artery
RITAD: Diameter of the postoperative right internal thoracic artery graft
RA: Radial artery
RAD: Diameter of the radial artery
RAD1: Diameter of the radial artery before the first anastomosis
RAD2: Diameter of the radial artery before the second anastomosis
RITA: Right internal thoracic artery
SVG: Saphenous vein graft
